# Automated classification of dolphin echolocation click types from the Gulf of Mexico

**DOI:** 10.1371/journal.pcbi.1005823

**Published:** 2017-12-07

**Authors:** Kaitlin E. Frasier, Marie A. Roch, Melissa S. Soldevilla, Sean M. Wiggins, Lance P. Garrison, John A. Hildebrand

**Affiliations:** 1 Scripps Institution of Oceanography, La Jolla, California, United States of America; 2 San Diego State University, San Diego, California, United States of America; 3 NOAA NMFS Southeast Fisheries Science Center, Protected Resources and Biodiversity Division, Miami, Florida, United States of America; CNRS -LSIS USTV, FRANCE

## Abstract

Delphinids produce large numbers of short duration, broadband echolocation clicks which may be useful for species classification in passive acoustic monitoring efforts. A challenge in echolocation click classification is to overcome the many sources of variability to recognize underlying patterns across many detections. An automated unsupervised network-based classification method was developed to simulate the approach a human analyst uses when categorizing click types: Clusters of similar clicks were identified by incorporating multiple click characteristics (spectral shape and inter-click interval distributions) to distinguish within-type from between-type variation, and identify distinct, persistent click types. Once click types were established, an algorithm for classifying novel detections using existing clusters was tested. The automated classification method was applied to a dataset of 52 million clicks detected across five monitoring sites over two years in the Gulf of Mexico (GOM). Seven distinct click types were identified, one of which is known to be associated with an acoustically identifiable delphinid (Risso’s dolphin) and six of which are not yet identified. All types occurred at multiple monitoring locations, but the relative occurrence of types varied, particularly between continental shelf and slope locations. Automatically-identified click types from autonomous seafloor recorders without verifiable species identification were compared with clicks detected on sea-surface towed hydrophone arrays in the presence of visually identified delphinid species. These comparisons suggest potential species identities for the animals producing some echolocation click types. The network-based classification method presented here is effective for rapid, unsupervised delphinid click classification across large datasets in which the click types may not be known *a priori*.

## Author summary

Health of marine mammal populations is often considered an indicator of overall marine ecosystem health and resilience, particularly in highly-impacted regions such as the Gulf of Mexico. Marine mammal populations are difficult to monitor given the many challenges of observing animals at sea (e.g. weather, limited daylight, ocean conditions, and expense). An increasingly common approach is the use of underwater acoustic sensors capable of recording marine mammal calls at remote locations for months at a time. Acoustic sensors generate large datasets in which dolphin echolocation clicks are commonly present. Dolphins are the most diverse family of marine mammals, and distinguishing click characteristics have only been described for a small subset of species. We developed a workflow to automatically identify distinct dolphin click types within large datasets without prior knowledge of their distinguishing features. Our algorithm then recognizes these click types in novel recording data across a range of monitoring locations. Known species-specific click types emerge from the data using this approach, as well as new click types potentially associated with additional species. This technique is a key step toward determining species identification for passive acoustic monitoring of offshore populations of dolphins and other toothed whales under a big data paradigm.

## Introduction

Dolphins produce echolocation clicks while socializing, foraging and traveling [1]. The prevalence of echolocation clicks makes these signals useful for monitoring delphinids using passive acoustic methods; however, only a few delphinid click types can currently be classified to species. Echolocation clicks have a suite of characteristics that make them challenging to classify in acoustic recordings. For example, echolocation clicks are highly directional signals which can be received “on-axis” (animal oriented in the direction of the recording sensor while clicking) or “off-axis” (animal oriented away from the sensor), leading to differences in amplitude and interference patterns [2]. Dolphin echolocation click signals also significantly attenuate over relatively short distances due to their high frequency acoustic content; therefore, the orientation and proximity of a clicking animal relative to an acoustic sensor has a large effect on the frequency structure of the recorded click [3, 4]. Behaviorally, individual dolphins may vary click source levels and beam widths [5–8]. Furthermore, dolphins are typically found in large, sometimes multi-species groups in which animals vocalize simultaneously. All of these factors contribute to click variability and therefore complexity in classification.

Despite these sources of variability, echolocation clicks of a few delphinid species as well as many beaked whale species have known species-specific spectral features [9–12]. Consistent features have typically been recognized by expert analysts manually reviewing large amounts of data. Previously identified characteristic spectral features include mean frequency, bandwidth, and peaks or troughs in frequency spectra indicating dominant or diminished frequencies. Typical inter-click interval (ICI) ranges also differ between beaked whale species [13], and ICI is used to identify porpoise click trains to species [14, 15], although ICI may vary as a function of depth or behavior in some cases [1, 16, 17].

A challenge in echolocation click classification is to overcome the many sources of variability to recognize underlying consistent patterns. One approach is to train analysts to recognize patterns. Humans are particularly adept at pattern recognition tasks: With enough training time, contextual information and training data, an analyst can distinguish within-type and between-type click variations, and develop a sense of the major click categories in a dataset. However this is an iterative, time-consuming and potentially subjective process.

An alternative is to develop automated methods to perform echolocation click classification. Within a computational framework, one approach to the click variability problem is to consider a set of clicks as a group of objects that are similar but not identical to one another. In a simple example with five clicks labeled A through E, consider a case where clicks A, B and C are very similar, click D is slightly different, and click E is very different than A-C, with some similarity to D. In this case, clicks A, B and C are regarded as the most informative for classification, as they contain consistent features among them, while clicks D and E are likely outliers. We might consider A, B and C to be members of a group characterized by their common feature set. In practice, an actively echolocating dolphin produces multiple clicks per second. Therefore, a similar but more complex case exists in which a subset of those clicks will be highly interrelated, while others are only weakly associated.

**Fig 1:**
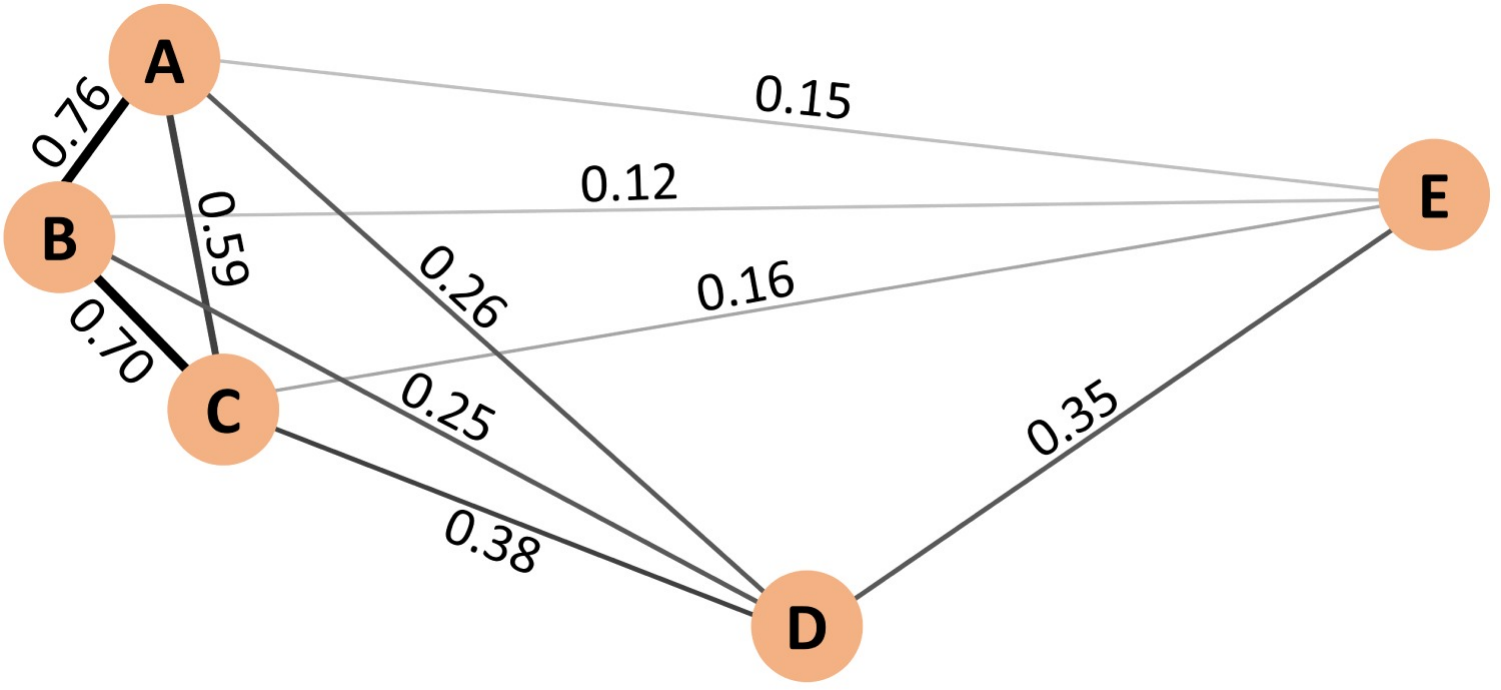
Example network representing relationships between echolocation clicks A-E. Circles represent nodes and lines are edges representing the similarity between nodes. Edge weight values indicate the similarity between each pair of connected nodes; where 1 indicates that the two nodes are identical, and 0 indicates that they are unrelated. This network is synthetic for illustrative purposes.

This approach to the variability problem can be represented as a weighted network [18], in which clicks are represented by nodes and the lines or edges between nodes represent the strength of the similarity between them. In the example above of echolocation clicks A through E, the click characteristic inter-relationships are represented by a network with larger edge weights among similar clicks A-C and lower value edge weights among clicks D and E and their neighbors which show their greater dissimilarity from clicks A-C and each other (Fig 1). A network of *N* nodes can also be represented as an adjacency matrix *G* in which *G*(*i*,*j*) represents the weight of the edge between nodes *i* and *j*, for *i* and *j* ∈ the set of nodes *N* [19].

Once the relationships between a set of clicks are represented as a network, an unsupervised learning algorithm can be used to identify clusters of highly similar clicks. Here we use an agglomerative clustering routine [20] that seeks to identify structure within the network without *a priori* information about what that structure might be. Using this method, nodes within the network are iteratively grouped together based on the strengths of the edges between them. This method can converge to a single large cluster if all nodes are highly interrelated, but multiple clusters can be identified if interrelationships are not evenly spread across the network.

In this work, unsupervised network-based classification methods are applied to the problem of delphinid echolocation click classification in the Gulf of Mexico (GOM). Long-term passive acoustic monitoring efforts using autonomous near-seafloor hydrophones at five sites in the GOM have resulted in a dataset of over 52 million unlabeled dolphin echolocation clicks. Thirteen delphinid species are known to inhabit the GOM, including five members of the genus *Stenella*, and five species belonging to the subfamily Globicephalinae (Table 1). Three of these five species, Risso’s dolphin (*Grampus griseus*), false killer whale (*Pseudorca crassidens*) and short-finned pilot whale (*Globicephala macrorynchus*) can be distinguished based on echolocation click characteristics [11, 21]; however, few other species have been conclusively identified.

**Table 1:** Gulf of Mexico delphinids listed in order of estimated abundance according to NOAA stock assessments [22, 23].

Common Name	Latin Name
Common bottlenose dolphin	*Tursiops truncatus*
Pantropical spotted dolphin	*Stenella attenuata*
Atlantic spotted dolphin	*Stenella frontalis*
Spinner dolphin	*Stenella longirostris*
Short-finned pilot whale	*Globicephala macrorhynchus*
Melon-headed whale	*Peponocephala electra*
Risso’s dolphin	*Grampus griseus*
Striped dolphin	*Stenella coeruleoalba*
False killer whale	*Pseudorca crassidens*
Rough-toothed dolphin	*Steno bredanensis*
Clymene dolphin	*Stenella clymene*
Pygmy killer whale	*Feresa attenuata*
Killer whale	*Orcinus orca*
Fraser’s dolphin	*Lagenodelphis hosei*

Our objectives are to develop a technique for recognizing candidate click types in this dataset which may be associated with species that are not yet acoustically identifiable, and to demonstrate a method for recognizing these click types automatically in novel data. Further, we support the utility of this method by comparing automatically identified types with clicks recorded using towed hydrophone arrays in the presence of vocalizing animals from the western Atlantic whose species identity has been verified by trained visual observers. The described click types are informative for passive acoustic delphinid population monitoring efforts, while the methods offer an approach for automated classification of variable signals in large unlabeled acoustic datasets.

## Results

### Click detection

Long term passive acoustic recordings were collected at three continental slope sites (sites MC, GC, and DT), and two shelf sites (sites DC and MP). Delphinid clicks were automatically detected in large numbers during all deployments at each site, with click counts ranging from 5.2x10^5^ to over 8.1x10^6^ analyst-confirmed detections per deployment (between 6,000 and 67,000 clicks per day; Table 2). Detections were grouped into 5-minute bins marked as click-positive or negative. The number of click-positive 5-minute bins per deployment varied from almost 5,000 to close to 12,000 bins (unnormalized for recording effort). The average number of delphinid echolocation encounters (periods of continuous click detections bounded before and after by at least 15 minutes without click detections) per recording day ranged from 1.4 to 7.9 across deployments. Average encounter durations were generally shorter at the shelf sites MP and DC; however, encounter durations were highly variable at all sites and ranged from 1 to 640 minutes. Across all deployments, between 0.1% and 10.1% of click-positive bins contained more than 5000 clicks and were sub-sampled for classification purposes. The most sub-sampled site was site DT.

**Table 2:** Gulf of Mexico HARP training and testing set deployment periods, locations, and automated click detector results. Site designations are: MC = Mississippi Canyon, GC = Green Canyon, DT = Dry Tortugas, DC = DeSoto Canyon, and MP = Main Pass. Counts and durations of click detections and encounters were computed after false positive detections had been removed. Five minute bins containing > = 100 click detections were included in the classification analysis. Bins containing more than 5000 clicks were subsampled. Bold rows indicate deployments used for classifier testing; all other rows represent deployments used for classifier training.

Data ID	Site Long. W	Site Lat. N	Site Depth (m)	Data Start (mm/dd/yyyy)	Data End (mm/dd/yyyy)	Recording Duration (days)	Clicks Detected	Click Encou-nters	Encounter Duration (minutes) Mean (CV)	5-min Bins > = 100 clicks	% of Bins >5000 clicks	Clusters per bin Mean (CV)
MC01	88^o^ 27.93’	28^o^ 50.75’	980	05/16/2010	08/28/2010	104	4,098,257	290	66.4 (1.08)	2,213	9.1	1.14 (0.35)
MC02	88^o^ 27.91’	28^o^ 50.77’	980	09/07/2010	12/19/2010	103	3,938,392	326	57.3 (1.13)	2,016	9.1	1.13 (0.35)
**MC03**	**88**^**o**^ **27.91’**	**28**^**o**^ **50.78’**	**980**	**12/20/2010**	**03/21/2011**	**91**	**2,459,669**	**302**	**56.7 (1.27)**	**1,792**	**5.3**	**1.08 (0.30)**
GC01	91^o^ 10.01’	27^o^ 33.47’	1,115	07/15/2010	10/11/2010	88	2,536,849	247	57.6 (1.01)	1,669	5.5	1.14 (0.34)
GC02	91^o^ 10.01’	27^o^ 33.47’	1,160	11/08/2010	02/02/2011	86	768,724	123	44.3 (0.75)	547	4.9	1.05 (0.22)
**GC03**	**91**^**o**^ **10.07’**	**27**^**o**^ **33.42’**	**1,100**	**03/23/2011**	**08/08/2011**	**138**	**4,400,383**	**502**	**54.5 (1.09)**	**2,885**	**6.3**	**1.06 (0.26)**
DT01	84^o^ 38.25’	25^o^ 31.91’	1,320	08/09/2010	10/26/2010	78	5,178,074	291	80.3 (1.09)	3,005	8.1	1.02 (0.14)
DT02	84^o^ 38.25’	25^o^ 31.91’	1,320	03/03/2011	07/12/2011	129	6,986,199	403	84.1 (1.16)	4,236	7.8	1.02 (0.14)
**DT03**	**84**^**o**^ **38.26’**	**25**^**o**^ **31.86’**	**1,300**	**07/13/2011**	**11/14/2011**	**124**	**8,124,736**	**383**	**107.3 (2.19)**	**4,666**	**9.0**	**1.02 (0.14)**
DC02	86^o^ 05.77’	29^o^ 03.13’	268	10/21/2010	02/06/2011	108	4,721,267	849	30.7 (1.56)	2,162	10.1	1.07 (0.27)
DC03	86^o^ 05.80’	29^o^ 03.21’	260	03/21/2011	07/06/2011	107	1,951,751	828	34.2 (1.50)	2,128	2.4	1.03 (0.15)
**DC04**	**86**^**o**^ **05.90’**	**29**^**o**^ **02.89’**	**260**	**10/26/2011**	**03/02/2012**	**144**	**4,137,213**	**607**	**35.6 (0.97)**	**2,079**	**9.3**	**1.04 (0.22)**
MP01	88^o^ 17.53’	29^o^ 15.20’	86	07/04/2010	09/10/2010	68	526,293	114	37.5 (1. 09)	736	0.1	1.08 (0.35)
MP02	88^o^ 17.81’	29^o^ 15.32’	93	11/07/2010	02/19/2011	100	2,079,315	242	91.9 (2.06)	2,500	2.5	1.06 (0.29)
**MP04**	**88**^**o**^ **17.70’**	**29**^**o**^ **15.35’**	**93**	**09/22/2011**	**03/01/2012**	**161**	**989,293**	**387**	**42.3 (1.16)**	**1,654**	**0.8**	**1.06 (0.24)**

### Click type identification

#### Exploratory analysis

**Fig 2:**
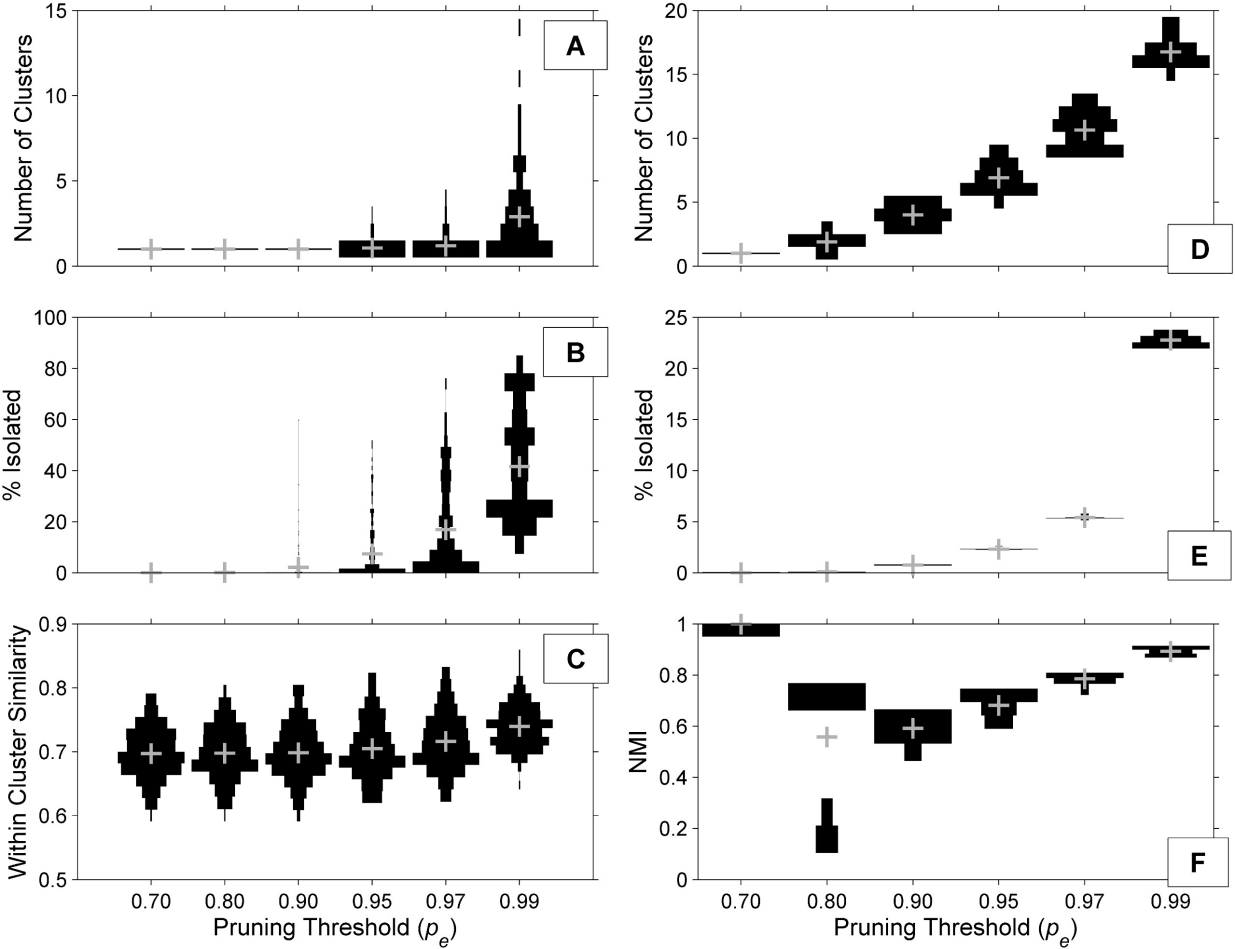
Effect of edge pruning threshold (*p*_*e*_). The effects of *p*_*e*_ choice on Phase 1 clusters (subplots A-C) were evaluated on a subset of bins from site MC. Effects on Phase 2 clusters (subplots D-F) were evaluated by running 20 iterations of the CW routine at each threshold. Horizontal bars represent the normalized distributions of each measured parameter, with the gray + indicating the parameter mean. In both phases, increasing *p*_*e*_ increased the mean number of clusters identified and the mean percentage of nodes. Measures of cluster purity (Phase 1: within-cluster similarity, Phase 2: NMI) also generally increased, with the exception of *p*_*e*_ = 0.70 (NMI = 1 with a single cluster across all partitions and no isolation). A mid-range threshold of *p*_*e*_ = 0.95 was selected for both phases to allow identification of multiple clusters without over-pruning or over-training.

The automated network-based clustering analysis used a two-phase process to identify candidate click types in the training dataset. An exploratory analysis examining the effects of a user-defined edge pruning parameter *p_e_* was conducted using a range of *p_e_* values. In Phase 1 clustering was performed on the spectra of echolocation clicks in five-minute bins, with *p_e_* controlling the percentage of weakest edges in the network to be removed. Removing ≤ 90% of the edges (*p_e_* = 0.90) resulted in one cluster per bin, across all bins, with less than 0.2% of nodes isolated on average (Fig 2A–2C). As *p_e_* increased, the mean number of distinct clusters identified per bin, percentage of nodes isolated, and within cluster similarity increased. High within-cluster similarity indicates well-defined clusters, while high node isolation excludes data and large numbers of clusters may indicate over-training, therefore an intermediate threshold is needed. A *p_e_* threshold of 0.95 increased the mean number of clusters per bin to 1.1, such that 5.7% of bins contained more than one cluster, and an average of 7.4% of nodes were isolated. At *p_e_* = 0.99, 64.4% of bins contained more than one cluster. Based on a manual review of the data, a small but significant number of time bins contained more than one click type, but more than two were uncommon, therefore a mid-range pe threshold of 0.95 was used for the Phase 1 clustering step.

Phase 2 clustering was performed on the summary spectra and ICI distributions (summary nodes) resulting from Phase 1. In the exploratory analysis, *p*_*e*_ ≤ 0.70 or less resulted in a single cluster across 20 iterations of the Chinese whispers (CW) algorithm, with zero isolated nodes. Cluster consistency, quantified as the mean normalized mutual information (NMI) between pairs of partitions across multiple trials resulted in mean NMI of 1 (CV = 0) for *p*_*e*_ ≤ 0.70 (Fig 2D–2F). A *p*_*e*_ threshold of 0.80 yielded 1.9 clusters on average across 20 iterations, isolated 0.09% of nodes, and produced highly variable, bimodal mean NMI score, suggesting unstable partitions. As *p*_*e*_ increased from 0.80 the number of clusters identified, number of isolated nodes, and mean NMI grew. NMI variability decreased, suggesting more stable partitions at higher pruning thresholds, likely because outlier summary nodes had been isolated from the network. The strongest *p*_*e*_ threshold tested (0.99) isolated nearly 25% of the nodes, and identified 16 clusters on average, many of which were small (fewer than 50 nodes) or duplicates (very similar spectra and ICI distributions to other clusters). The exploratory analysis suggested that a *p*_*e*_ value of 0.95 led to stable partitions with minimal isolation and few overly-trained or duplicate clusters.

### Click types

**Fig 3:**
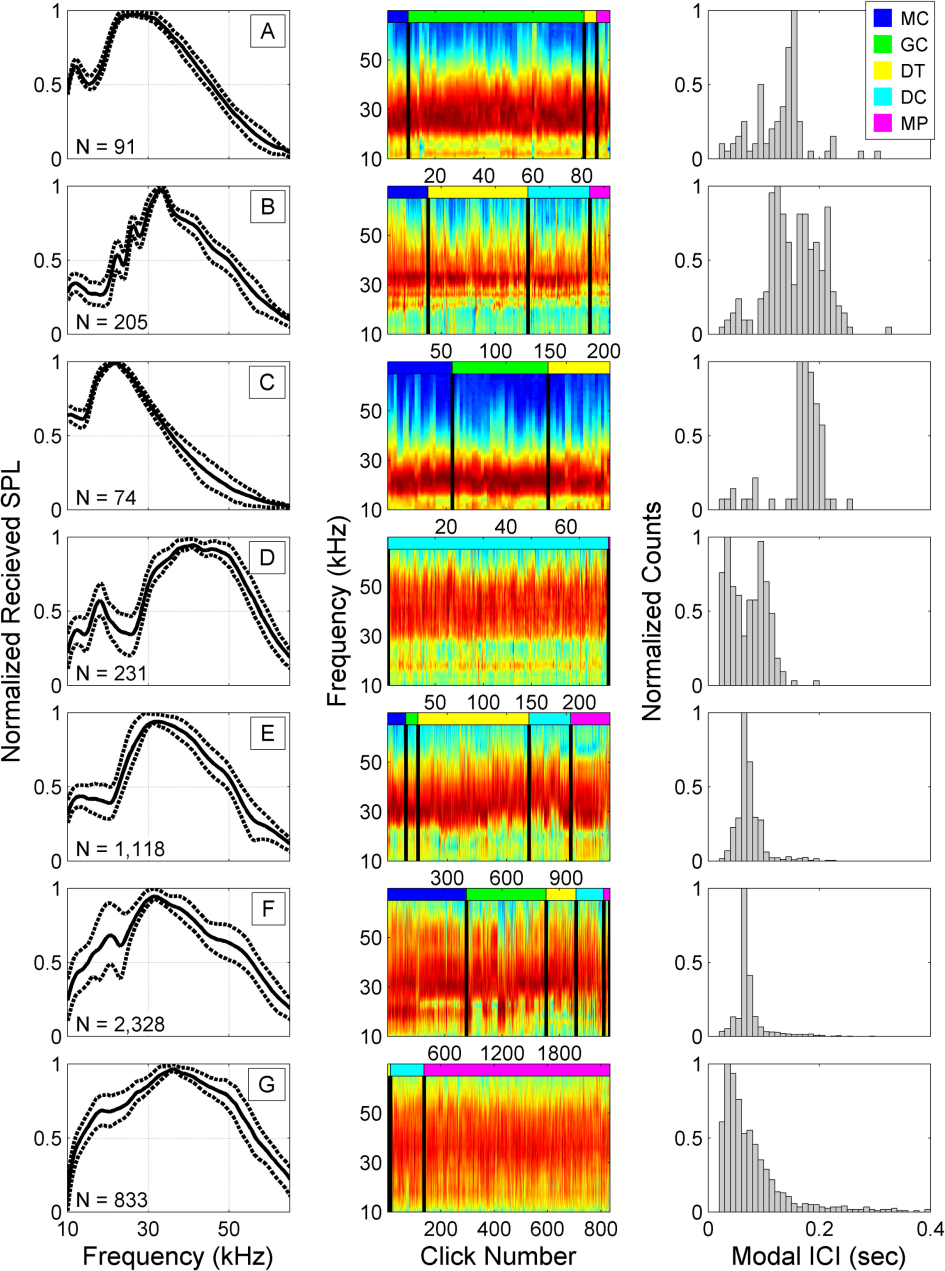
**Normalized sound pressure level (SPL) spectra, concatenated spectra, and ICI distributions of the seven dominant click types (A-G) identified in the training datasets across five sites by the automated clustering routine.** Each row of plots represents a distinct cluster in the final partition. *Left*: Normalized mean cluster received sound pressure level spectra (solid line) with 25^th^ and 75^th^ percentiles (dashed lines). N indicates the number of summary nodes included in each cluster. *Center*: Concatenated mean spectra of the summary nodes in each cluster. Color scale indicates relative amplitude in dB (red is high, blue is low). Colored bar across the top indicates the site from which the spectra below were extracted. Site/color pairs are: MC/Blue, GC/Green, DT/Yellow, DC/Cyan, MP/Magenta. *Right*: Distribution of modal ICIs.

Phases 1 and 2 were run on the full training set following the exploratory analysis. In Phase 1, the average number of automatically identified clusters per time bin ranged from 1.02 to 1.14 (CV = 0.14 and 0.35 respectively) across sites and deployments (Table 2). In Phase 2, seven dominant and recurrent click types (A-G) characterized by consistent spectral shapes and modal ICIs were identified (Table 3, Fig 3). We define the modal ICI as the most frequently observed ICI during a period of clicking.

**Table 3:** Frequency and ICI statistics by click type. Cluster size indicates the number of five-minute bins included in each click type cluster out of a set of 5,000 bins used for training. The mean of the modal (most frequently observed) ICI is computed across all five-minute bins in the cluster.

Click Type	Cluster Size (N)	Peak Frequency (kHz) Mean (CV)	Modal ICI (sec)	Mean of Modal ICI (sec) Mean (CV)
**A**	91	27.1 (0.10)	0.155	0.130 (0.38)
**B**	205	33.2 (0.07)	0.125	0.156 (0.32)
**C**	74	21.7 (0.06)	0.165	0.168 (0.27)
**D**	231	42.2 (0.10)	0.035	0.071 (0.46)
**E**	1,118	32.3 (0.11)	0.065	0.074 (0.33)
**F**	2,328	30.3 (0.18)	0.065	0.073 (0.37)
**G**	833	36.5 (0.16)	0.035	0.079 (0.76)

Click type A was identified in the training data from the three deep sites, and one shallow site. Most instances came from site GC. This type was characterized by a minor narrow low frequency peak near 12 kHz, dominant energy between 20 and 35 kHz, and 0.15 sec modal ICI.

Click type B was identified in the training data from all sites except site GC. This click type, presumed to be Risso’s dolphin based on Soldevilla *et al*. [11] and has distinct narrow energy peaks at ~ 22, 26, and 33 kHz. The ICI distribution for this type was bimodal with shorter ICIs near 0.12 sec at the northern sites, and longer ICIs over 0.23 sec at the southern site DT.

Click type C was identified in the training data from the deep sites only. This click type had the lowest frequency content of dominant energy between ~15 and 30 kHz, and a modal ICI of 0.16 sec.

Click type D was identified in the training data from site DC, and in one bin from site MP. This click type was characterized by two low frequency peaks at 12 and 18 kHz, dominant energy between 30 and 50 kHz, and a bimodal modal ICI with peaks at 0.03 and 0.09 sec.

Click type E was identified in the training data from all five sites and represented 22% of the training set. It was particularly common at the southern site DT. Click type E was characterized by minimal energy below 20 kHz, a dominant spectral peak near 30 kHz, and a modal ICI of 0.06 sec. Spectral variability below 20 kHz may indicate the presence of multiple subtypes, or overlap with click type F.

Click type F was identified in the training data from all five sites and represented 47% of the training set. This type was similar to type E, had a minor energy peak at approximately 20 kHz. Some examples had a third peak at 16 kHz. High variability of this type in the 10–25 kHz band suggests that it may include multiple subtypes. This type had a strong modal ICI at 0.06 sec.

Click type G was only identified in the training data from the two shallow sites only: Sites DC and MP. It was characterized by a broad high energy band between 15 and 52 kHz, and a peak frequency of 36 kHz and a modal ICI of 0.03 sec.

#### Cluster-based classification

Phase 1 clustering was conducted on the test data to produce summary nodes for each bin in the test set. The test nodes were then classified by automatically assigning them to one of the seven click types identified in the training set, based on similarity. The similarity score between each test node and its matching click type cluster was retained as a metric of classification certainty.

**Fig 4:**
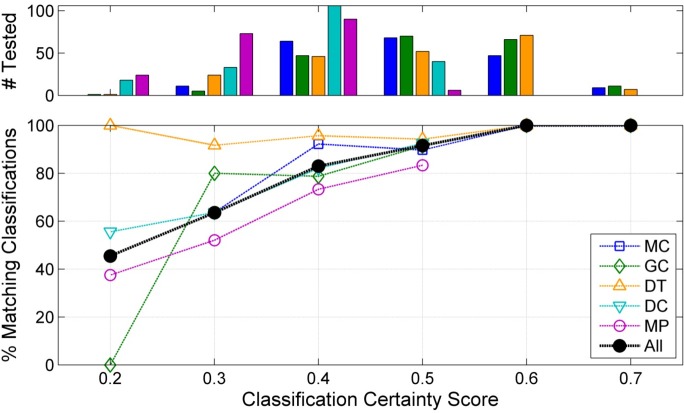
Classification agreement between automated and manual classifications as a function of classification certainty score. **Upper plot:** Number of bins tested manually per site at each classification certainty level. Site/color pairs follow the legend in the lower right plot. **Lower plot:** Percentage of matching automated and manual classifications as a function of classification certainty score based on a comparison with a subset of 200 manually classified test bins per site. Black line represents the percentage of matching classifications between the two methods across all sites combined.

To evaluate the classifier performance, an analyst-based manual review was conducted on a subset of the automated classifications. Analysts matched the summary nodes to the click type clusters obtained in Phase 2 based on normalized mean spectra and ICI distributions. This comparison indicated that classification certainty scores were a useful predictor of automated classification accuracy, and that both classification certainty and classifier performance varied within and between sites (Fig 4). Automated and manual classifications were in agreement for over 90% of test nodes across all sites when classification certainty scores were > = 0.5. Automated and manual classifications were in agreement for less than 60% of test nodes across all sites when classification certainty scores were < = 0.3. Classification certainty scores and classification accuracy were lowest overall at the shallowest site MP, due to high levels of contamination from false positives associated with snapping shrimp. Based on this analysis, test click types with match certainty scores below 0.3 were classified as unknown.

Click type C was predominantly found at deep sites (MC, GC and DT), and click types D and G were predominantly found at shallow sites (DC and MP), as found in the training dataset (Table 4). The most common classifications assigned to the test set were types E and F, as found for the training data.

**Table 4:** Rates of occurrence of each click type (A-G) in test sets by site. Numbers indicate the percentage of test click types assigned to each training cluster by the automated classification algorithm for each site. Types that were predominantly restricted to shallow sites (MP and DC) in the training dataset were infrequently identified at deep sites during testing, and vice versa.

	A	B	C	D	E	F	G	Unknown	Total
**MC**	2.2	17.3	1.5	0.4	19.2	56.1	0.9	2.5	100
**GC**	8.1	1.8	4.1	0.2	15.8	68.2	0.3	1.4	100
**DT**	0.8	24.5	0.4	0.2	52.8	19.2	0.8	1.3	100
**DC**	1.7	0.2	0.1	23.8	32.7	12.6	19.2	9.7	100
**MP**	4.4	0.2	0.0	1.9	30.9	4.2	44.4	14.0	100
**All Sites**	3.2	12.3	1.3	4.0	34.4	33.5	7.4	3.9	100

Classifier confusion was evaluated by comparing the automated and manual classifications in the manually verified test set (Table 5). The most common source of confusion was a mismatch between auto-classifications of type E and manual classifications of types D or G. Over 46% of the mismatches were associated with site MP, where snapping shrimp false positive contamination of summary nodes likely reduced match quality. Across all sites, the analyst was more likely to label test click types as unknown than the automatic classifier: 47% of mismatched classifications were cases where the automated classifier identified a matching template, while the analyst left the type unknown.

**Table 5:** Classifier confusion across all sites based on manual evaluation. Rows indicate automatic classifications and columns indicate manual classifications, for a manually verified subset of 1000 test summary nodes across five sites. Values represent the number of instances of each combination. Values on the diagonal indicate cases of agreement between the two classification methods. “Unk” labels represent test click types that were labeled as unknown because match confidence was low (automatic classification) or because they did not clearly match a template cluster (manual classification).

		Manual Classification							
		*A*	*B*	*C*	*D*	*E*	*F*	*G*	*Unk*.
**Automatic Classification**	***A***	**12**	1	0	0	2	4	2	14
	***B***	0	**88**	0	0	0	0	0	0
	***C***	0	0	**12**	0	0	0	0	0
	***D***	0	0	0	**44**	0	0	0	3
	***E***	0	1	0	18	**259**	3	14	12
	***F***	0	0	7	0	0	**291**	4	19
	***G***	0	0	1	2	0	0	**108**	27
	***Unk*.**	0	2	0	12	2	0	8	**28**

#### Towed hydrophone array recordings

**Fig 5:**
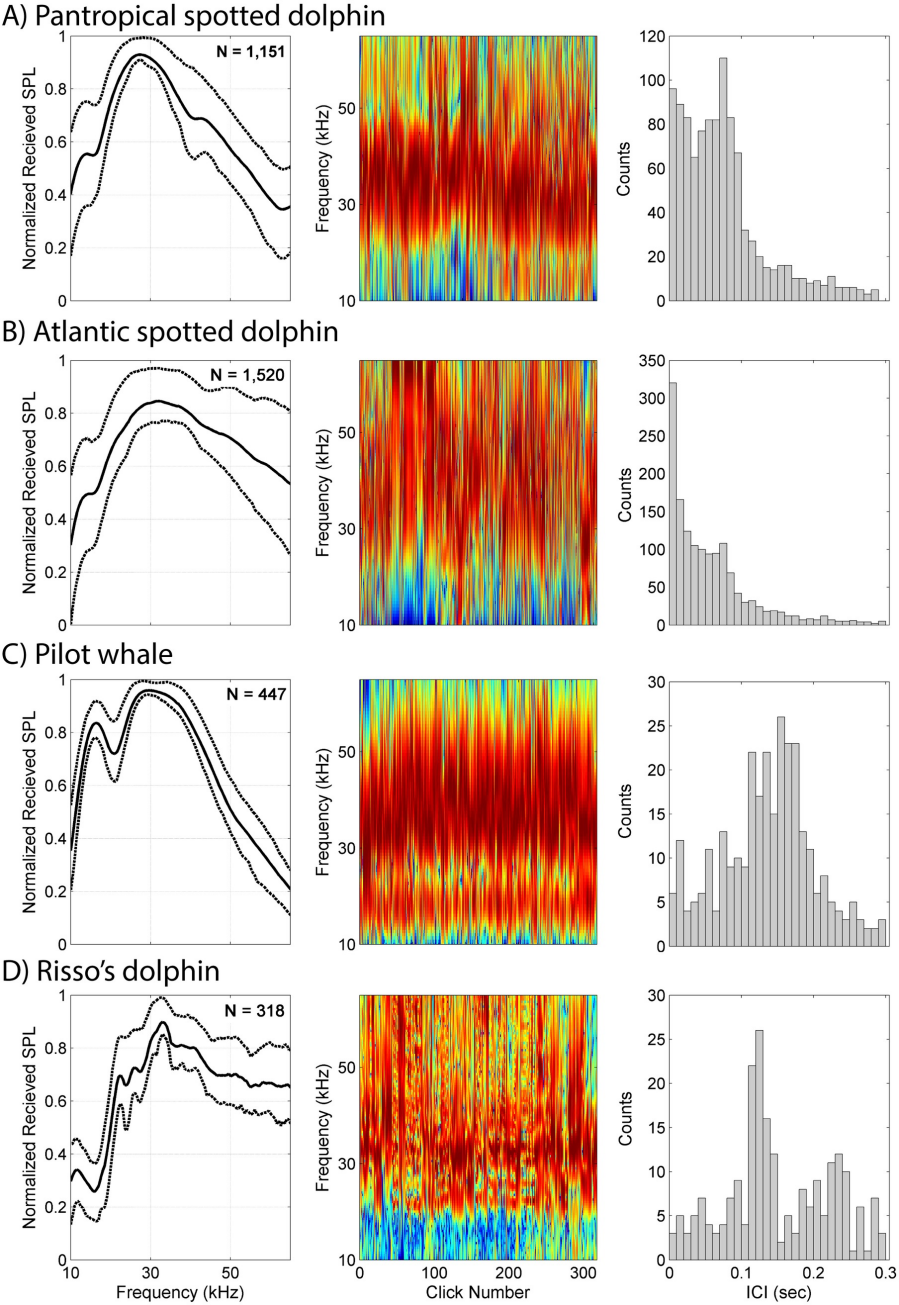
Towed hydrophone array click types. Clustered clicks from towed hydrophone array recorded in the presence of (A) Pantropical spotted dolphin (*Stenella attenuata*), (B) Atlantic spotted dolphin (*Stenella frontalis*), (C) Pilot whale (*Globicephala sp*.), and (D) Risso’s dolphin, (*Grampus griseus*). B, C and D were recorded in the Atlantic. *Left subplots*: Mean normalized spectra levels (solid line) with 25^th^ and 75^th^ percentiles (dashed lines); *n* indicates the number of clicks retained in the final cluster. *Center subplots*: Concatenated spectra of all clustered clicks. Color indicates normalized received sound pressure level (SPL). *Right subplots*: Inter-click interval (ICI) distributions.

Preliminary characteristic click type features (mean normalized spectral levels and ICI distributions) were identified from towed hydrophone array recordings for pantropical spotted dolphin, Atlantic spotted dolphin, pilot whale (presumed short-finned), and Risso’s dolphin (Fig 5). Pantropical and Atlantic spotted dolphin clicks (Fig 5A and 5B) had modal ICIs at 0.075 sec, similar to type E and F clicks. In the case of Atlantic spotted dolphin (Fig 5B) the modal ICI is weak, masked by high counts at lower ICIs, possibly due to overlapping click trains. Pilot whale clicks had lower frequency distributions than the spotted dolphin clicks, and a modal ICI of 0.155 sec (Fig 5C). These clicks are most similar in spectra and ICI to type A clicks; however, the location of the low frequency secondary peak differs between the two. Risso’s dolphin clicks from the towed array data had a modal ICI of 0.12 sec (Fig 5D) and frequency peaks at 22, 26, 30.5 and 33 kHz, those described by Soldevilla *et al*. [11] and type B clicks from the training set.

## Discussion

### Automatic implementation choices

Delphinid clicks are very short duration, highly variable signals which contain limited information when considered individually. The automated clustering strategy was designed to mimic a human analyst by comparing large numbers of clicks to identify persistent features. Leveraging multiple sources of information such as spectral shape and ICI distributions across bins of similar clicks further facilitated pattern recognition and click type distinction. The two-step training process tackled the large dataset by reducing the number of comparisons necessary through use of filtered means and modes.

A variety of different pruning and clustering techniques were implemented during the algorithm development process. In the final implementation, edge pruning was executed using a dynamic metric in which the weakest N% of edges were pruned from each network. Using this approach, networks of highly similar nodes and networks of weakly similar nodes were pruned by the same amount. An alternate approach would be to prune all edges weaker than a static threshold value. Using the static approach, a network of weakly interrelated nodes would be pruned more heavily than a network of strongly interrelated nodes. Both approaches were tested during development of the clustering protocol, but the dynamic metric was ultimately chosen as the more conservative pruning method for preserving click types with smaller sample sizes. More aggressive pruning at site MP might reduce inclusion of false positives associated with snapping shrimp and improve classification accuracy if snap spectra are more variable than click spectra.

A more complex, greedy clustering algorithm [modularity; 24, 25], preliminarily used during the development process, was not able to reliably identify clusters of different sizes. The simpler CW algorithm used in the final implementation identified both small and large clusters within a network, which is essential in identifying less common click types. Further click type separation may be possible however. In this dataset, some click types had very different spectral shapes and ICIs from one another such as type A and B clicks, while others were similar, such as type E and F clicks. This is a challenging situation for clustering purposes, because some types separate well, while others remain intermingled, as in the case of types E and F where spectral variability may represent multiple sub-types. In Phase 2, a multi-pass clustering approach in which thresholds were incrementally increased might enable better distinction between similar types such as those within type E without over-pruning highly distinct types. Reduced within-cluster variability would probably also reduce classifier confusion and improve accuracy.

ICI and spectral similarities (both values between 0 and 1), were combined in Phase 2 of the automated classification process by simple multiplication. The multiplicative rule was used because analysts typically needed both robust ICI and spectral information to make a confident classification. The two metrics did not necessarily contribute equally to the overall similarity scores because although they are both values between [0,1], they did not have identical distributions. Transforming the distribution of either parameter prior to multiplication would modify the influence of the parameter on the Phase 2 network. For example, if spectra were deemed more reliable than ICI, *S*_*SPEC*_ could be transformed prior to Eq (2) to give it more influence on the network. For classification of the test set, the multiplication method requires that both score high to achieve a high overall similarity score.

An earlier implementation of this algorithm used correlation distance between ICI distributions instead of distance between modal ICIs. This strategy produced similar results but performance suffered when classifying bins with high click counts. As the number of detections per bin increased, click trains tended to become interleaved, resulting in higher numbers of low ICIs. While true ICIs from a single animal’s click train may be a species-specific feature [26], the interval between clicks received from multiple individuals’ trains is not informative. Similarly, high false positive rates associated with snapping shrimp at site MP affected ICI distributions. Modal ICI, which likely represents individuals’ ICIs, was found to be less sensitive to differences in click counts per bin and more robust to false positives. Modal ICI may be more difficult to detect for species that are often found in very large groups.

### Click types

The unsupervised click classification routine identified seven distinct delphinid click types in the training data across five sites in the Gulf of Mexico based on frequency content and modal ICI. All types were identified at a minimum of two sites, and over half were identified at four or more sites.

One hypothesis of what is driving the persistent features leading to the click type clusters is site-specific propagation and noise conditions; however, a number of features demonstrated here are inconsistent with this explanation. First, site-specific noise and propagation do not explain why multiple click types were found at each site, often within the same day or in overlapping encounters, nor do they explain why the same click types were found at multiple sites, despite differences in noise, site depth, and site location. Second, site-specific propagation and noise would be expected to affect all clicks in the same way; therefore, they do not explain why some click types have complex spectra with peaks and troughs, or why frequency distributions differ between types under similar noise conditions. Third, site-specific conditions do not offer an explanation for the consistent relationships between click type spectral shape and ICI distributions across deployments spanning multiple years, or why ICI distributions have consistent modal values.

Alternative hypotheses are that the distinct click types identified in this dataset represent different dolphin species or echolocation clicks used in different contexts [e.g. 27]. Species differences may explain these observations. Echolocation click frequency content and click rates have been shown to differ between odontocetes such as sperm whales, beaked whales, dolphins, and porpoises [e.g. 11, 12, 13, 28]; therefore, it is reasonable to expect that these features may also differ between delphinid genera and/or species. Consistent ICIs have been reported for beaked whale species [e.g. 13] and similar consistency may be typical of some delphinids [29]. Spectral content may vary depending on target prey [9], and ICI may be related to click source level, frequency content, and/or prey search distance [e.g. 30, 31]. Low frequency, high amplitude clicks have the potential to propagate farther than high frequency or low amplitude clicks. This may result in a longer two-way travel time for each click. Delphinids may compensate with a longer ICI to allow for the longer travel times.

The majority of clicks detected at the three deepest sites were associated with types E and F which had similar spectral shapes and modal ICIs. According to the most recent NOAA stock assessments [22, 23] based on summer visual surveys, approximately 80% of offshore delphinids in the GOM are members of the *Stenella* genus, of which spinner and pantropical spotted dolphins are the most common species. Two additional *Stenellid* species, striped and Clymene dolphins, are also found offshore, although population estimates vary widely between surveys. A fifth species, Atlantic spotted dolphin, is found primarily on the continental shelf. Based on the high abundance of *Stenellids* as a proportion of GOM delphinids, *Stenellid* dolphins are the most likely match for type E and F clicks. Considerable variability below 20 kHz within sites in the type E and F clusters suggests that they may include multiple subtypes, possibly representing different species. Towed hydrophone array recordings made in the presence of pantropical and Atlantic spotted dolphins revealed ICIs that were consistent with type E and F clicks. Distributions of the various *Stenellid* species differ in the GOM [32], and this may account for the different ratios of these types across sites.

Based on visual survey data, species composition and abundance is expected to differ between the three deeper slope sites (GC, MC, and DT) and two shallower shelf sites (MP and DC). Primary species at the shallow sites include Atlantic spotted dolphin (also a member of the genus *Stenella*) and bottlenose dolphin [32]. Rough-toothed dolphins have also been observed near site DC, although in much lower numbers. Click type G which was common at the two shallow sites but was not identified at deeper locations, and click type D which was predominantly identified at site DC, are likely associated with some of these species. Snapping shrimp snaps were a common source of false positives at site MP, where click type G was primarily detected. Distributions associated with this click type may have been contaminated by snap signals. In future work, click train tracking could be used to improve ICI estimates in noisy, shallow water environments, and encounters with very high click counts.

Click Type B likely represents Risso’s dolphin clicks as it contains the consistent peaks and notches described for Risso’s dolphins in the Southern California Bight, and further matches the peak structure documented here from a towed array recording of visually-verified Risso’s dolphins from the western Atlantic. Modal ICI differed between the three northern sites (MC, DC, and MP) and the southern site (DT), suggesting possible behavioral or population differences.

Click type A may represent short-finned pilot whale clicks as it is similar to Atlantic pilot whale (presumed short-finned) recordings collected using towed hydrophone arrays. However, it differs from a recent description of Pacific short-finned pilot whale clicks which found spectral peaks at 12 and 18 kHz collected in the Hawaiian Islands [21]. Click type A was most common at site GC in this dataset, which is consistent with short-finned pilot whales’ predominantly eastern GOM distribution based on visual surveys [32].

The narrower bandwidth of click type C centered at lower frequencies is consistent with published descriptions of false killer whale (*Pseudorca crassidens*) echolocation clicks [9, 21] from the Eastern Pacific. However, there are no published estimates of modal ICI for false killer whales. Across all sites, 1.3% of bins were classified as Type C. The most recent stock assessment estimates place false killer whales as approximately 1% of offshore GOM delphinids.

Melon-headed whales are expected in low densities the GOM, but information regarding distinguishing features of these clicks is limited [12], and no clear match was identified. Killer whale, pygmy killer whale and Fraser’s dolphin, although present in the GOM, may be too rare at these sites to be identified using these methods [23]. Use of a larger training set with a multi-pass strategy in which dominant types, such as E and F, were identified and removed could facilitate recognition of rare types.

### Cross-instrument comparisons

A subset of the identified click types had characteristics in common with clicks recorded in the presence of visually-identified species recorded using the towed hydrophone array. Unfortunately, with the exception of the pantropical spotted dolphin data, these recordings were collected in the Atlantic and can only be tentatively compared with GOM click types. Towed array hydrophones are typically much shallower than seafloor instruments, therefore the effect of acoustic propagation on recorded signals differs. Further work will seek to solidify and extend comparisons between seafloor sensor types and towed array recordings of known species, with an emphasis on collecting recordings of visually identified species in the GOM.

The towed array environment is different from that of the seafloor sensor. Towed array recordings are much more affected by vessel, ship-based electronic and wind-generated sea-surface noise, and shallow sound-speed profiles than autonomous seafloor recordings. The orientations of animals relative to the sensors differ between the two types of recordings, for example, during a ship survey, dolphins are often oriented toward the bow, while the sensor is towed behind the vessel; whereas seafloor instruments are located below dolphins traveling near the sea surface, and do not typically influence dolphin orientations. Animal behaviors likely differ as well because marine mammal surveys require daylight for visual marine mammal identification, but seafloor sensor recordings typically show that most delphinid clicks are detected at night [29]. In addition, comparisons of simultaneous towed array and HARP recordings in the GOM have shown that towed array detection ranges may be limited by signal refraction associated with warm surface mixed layer [33]. Towed array delphinid click recordings were often characterized by short encounters and highly variable click amplitudes. When animals were close enough to the towed array to be detectable, both on-axis (transmission beam oriented directly toward the sensor) and off-axis clicks were likely received, and on-axis clicks could be clipped due to high amplitudes at close range. In contrast, delphinid encounters recorded by near-seafloor HARPs were often longer in duration due to larger detection ranges. Click amplitudes tended to be lower, because delphinids were farther from the sensor, and off-axis clicks were less detectable according to click propagation simulations [34].

### Future developments

Several improvements could be made to the automated classification approach in future work. First, different distance metrics could be evaluated. In this study, a correlation distance metric was used to assess similarity between spectra as it was found to capture shape similarities more effectively than a simpler Euclidean distance. However, the correlation distance used assigns equal weight to all frequencies in the spectra, while high frequency amplitudes are expected to vary more than low frequencies because of acoustic attenuation. To account for this expectation, a weighted distance metric could be used that emphasizes low frequency shape. Alternatively, click shapes could be summarized as cepstra (inverse FFT of spectra, e.g. [28]) to emphasize particular aspects of overall shape. Preliminary experiments using cepstra and perceptual weighting were conducted as part of this study, however clustering results were poor. Nonetheless, more complex weighting strategies might be useful in future work.

Another improvement that could be considered is to evaluate the impact of pre-filtering spectra prior to classification. In this implementation, frequencies below 10 kHz were removed by a bandpass filter. Future classification efforts might benefit from including lower frequency spectral content. Recent work by Finneran *et al*. [4] suggests that delphinid clicks may have consistent spectral features below 10 kHz which may be useful for click classification [e.g. 21].

Improvements could also focus on using different metrics to capture persistent features of ICIs. In this study, clear modal ICI peaks were associated with the click types, and ICI previously has been found to be useful for classifying beaked whale clicks [13]. While delphinids have been shown to vary their ICI in wild and captive studies [1, 16], this typically occurs during terminal buzzes [35] which are much lower amplitude and occur less frequently than regular clicks [35, 36] and therefore, are much less likely to be detected in wild recordings [34]. Deep seafloor instruments (at depths of roughly 80 m or more) often receive only a single animal’s click train at a given time due to the animals’ narrow transmission beam patterns and distance from seafloor sensors; therefore ICI often is accurately calculated and modal ICI is representative of persistent features. On occasions when a group of animals is large and/or close to the sensor, multiple click trains will overlap and modal ICI values may become subject to saturation. Click train tracking [37] could be used to improve modal ICI estimates in saturated cases and in noisy or shallow environments.

Additional improvements could be made to fully automate the classification process. For example, false positives were manually removed from this dataset prior to classification. However, many sources of false positives, including beaked whales, sperm whales, and ships, have their own spectral and ICI characteristics. A similar approach to that described here could be used to build template clusters for false positive sources, allowing them to be identified and excluded automatically during classification. In addition to accelerating the analysis process, this approach could improve the removal of false positives within bouts of true detections (such as at shallow sites), facilitate false positive rate calculations, and provide certainty scores for removed detections. Future work will likely seek to combine clustering with deep learning methods as a possible refinement for improved classification.

Finally, future improvements should focus on evaluating sources of variability within click types and on linking distinct click types with delphinid species identity or behavior states. This work focused on identifying distinct click types, however, more work needs to be done to describe within-type variability. Delphinids have been shown to vary their clicks depending on context [e.g. 6, 16, 27]. The types described here are broad groupings, and are not meant to indicate a lack of variability within each type. Concurrent visual identifications with HARP recordings are needed to conclusively validate potential species associations. Future steps should include applying this method to a labeled dataset associated with visually-identified species to ground truth the approach. Continued development of unsupervised learning strategies for identifying consistent dolphin click types will advance marine mammal monitoring programs by facilitating delphinid and toothed whale species identification in data collected using autonomous passive acoustic sensors.

## Methods

### Data collection

**Fig 6:**
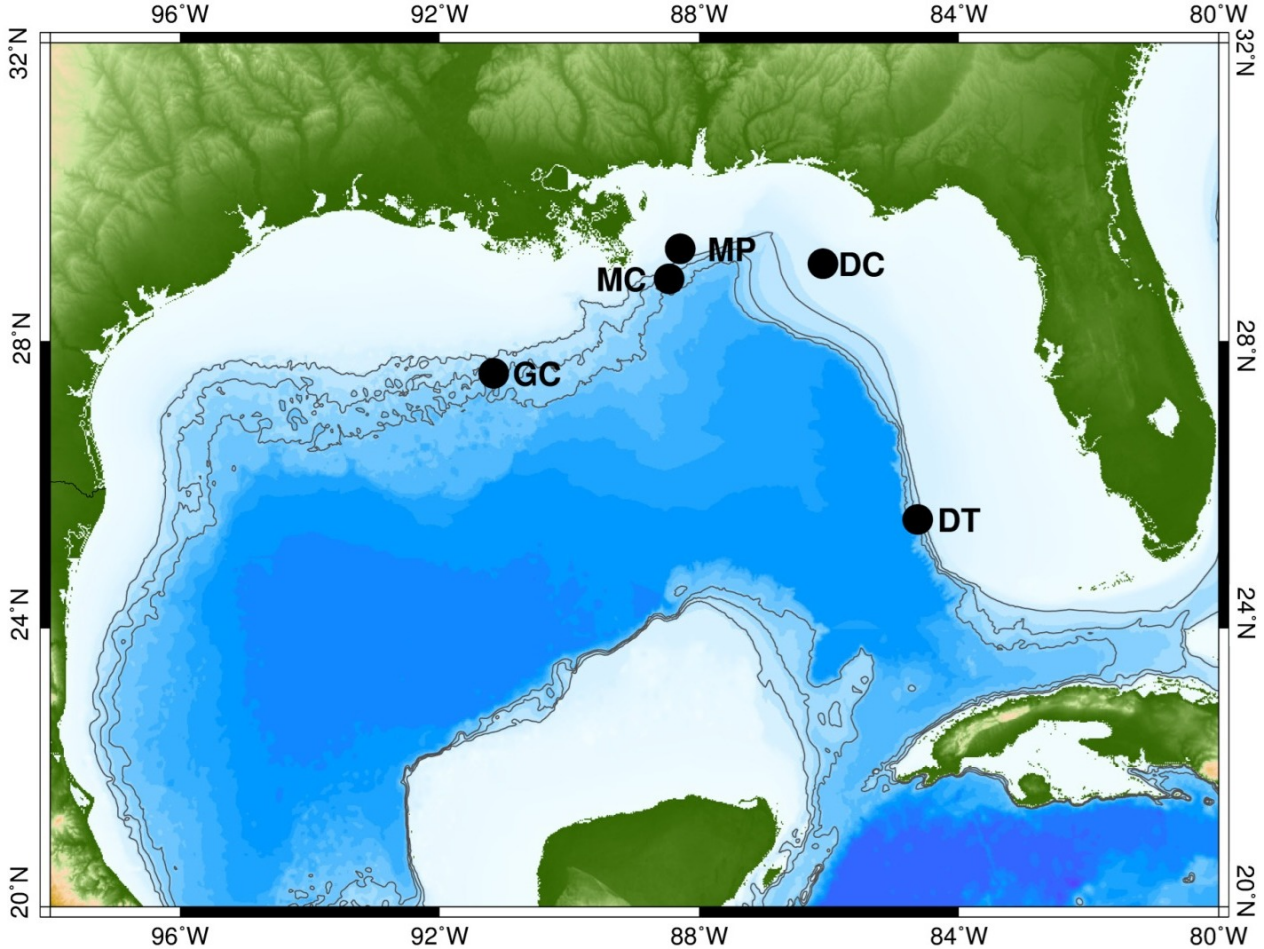
Map of recording site locations in the Gulf of Mexico with green/brown representing land masses, and white/blue representing water. The five sites are named based on the federal lease block in which they are located: Mississippi Canyon (MC), Green Canyon (GC), Dry Tortugas (DT), DeSoto Canyon (DC) and Main Pass (MP). Contours are at 500 m, 1000 m and 1500 m. Topographical data are from [38].

Long-term autonomous datasets were collected using High-frequency Acoustic Recording Packages (HARPs) at three continental slope and two shelf locations in the GOM between 2010 and 2012 (Fig 6). Details of each HARP deployment are presented in Table 2.

HARPs are autonomous bottom-mounted acoustic recorders containing a hydrophone, data logger, battery power supply, ballast weights, acoustic release system, and flotation [39]. All of the seafloor recording instruments used in this study were of the same type with equivalent hardware and software. Each instrument used a calibrated hydrophone (Channel Group Technologies, Santa Barbara, CA, ITC-1042) buoyed approximately 10 m above the seafloor. Hydrophones had an approximately flat (±2 dB) sensitivity from 10 to 100 kHz of -200 dB re V/μPa. Preamplifier calibrations were performed at Scripps Institution of Oceanography and at the U.S. Navy’s Transducer Evaluation Center facility in San Diego, California [38]. All HARPs sampled continuously at 200 kHz throughout each deployment.

Towed hydrophone array recordings were collected in 2011 and 2012 (Table 6) during National Oceanographic and Atmospheric Administration’s (NOAA) National Marine Fisheries Service (NMFS) Southeast Fisheries Science Center (SEFSC) marine mammal abundance surveys aboard the R/V Gordon Gunter, conducted in the eastern GOM and within the southeastern U.S. Atlantic coastal exclusive economic zone (EEZ). A team of visual observers identified dolphins to species whenever possible, thereby providing ground-truthed species identifications which acousticians could associate with concurrent array recordings. A five-element hydrophone array was towed 274 m behind the ship, at an estimated depth of 15 to 18 m at typical survey speed (10 kn). Hydrophone elements consisted of custom-built preamplifiers, with band-pass filters set for 3 dB roll-off at 1 kHz and 200 kHz, connected to an omni-directional spherical hydrophone (HS-150 Sonar Research and Development, Ltd., Beverley, UK). Two hydrophones separated by 2.12 m were sampled continuously at 500 kHz using a data acquisition board (NI USB 6251, National Instruments Corporation, Austin, TX) and recorded directly to hard disk drives using Logger 2000 (International Fund for Animal Welfare, IFAW, Yarmouth Port, MA). The towed array recording setup differs considerably from the seafloor sensors, therefore any comparisons are considered tentative.

**Table 6:** Towed hydrophone array recording locations, encounter dates, and click detection counts by species.

Species (common name)	Lat (N)	Lon (W)	Date (mm/dd/yyyy)	# of Clicks
Pantropical spotted	24 ^o^ 40.99’	85 ^o^ 09.76’	07/04/2012	1,228
Atlantic spotted	33 ^o^ 20.51’	77 ^o^ 11.57’	06/30/2011	1,673
Pilot whale (sp.)	37 ^o^ 16.05’	74 ^o^ 41.83’	07/14/2011	745
Risso’s dolphin	33 ^o^ 19.05’	76 ^o^ 35.26’	07/01/2011	563

### Data analysis

#### Detection

For acoustic detection and classification analyses, all acoustic data were band-pass filtered between 10 and 90 kHz. Echolocation clicks were detected using a simple energy detector [29] to identify impulse signals. Click start and end times were identified as the time when a 50 μsec smooth (moving average) of click energy fell below 95 dB re 1 μPa. Impulses with peak frequencies between 15 and 85 kHz, a high-energy envelope duration less than 10 μsec [Hilbert transform; 1, page 178], and received levels greater than 120 dB_pp_ re: 1μPa were retained as positive detections. Twenty samples before and after each detected click were included in the click time series. Click time series were Hann-weighted and zero-padded to 400 points. Spectra were computed for each detected click using a 400 point discrete Fourier transform (DFT) for a standard interpreted bandwidth of 50 Hz/frequency bin, and corrected for the hydrophone transfer function. Based on a tracking study [34], the detected clicks are far-field signals produced by dolphins at slant ranges up to approximately 2.5 km from the HARPs.

Large groups of false positive detections caused by ship passages, snapping shrimp, and non-target odontocete species (eg., sperm whales, pygmy sperm whales, and beaked whales) were removed manually by an analyst using *detEdit*, a custom graphical user-interface (GUI)-based tool [40] developed in MATLAB (Mathworks, Natick, MA) to ensure that retained signals were attributable to dolphins. Manual removal of false positives using this method is a rapid, batched process requiring only basic training compared to classification tasks.

#### Click type identification

Click detections from 15 HARP deployments was split into a training set (ten deployments, two per site) and a testing set (five deployments, one per site; Table 2). An unsupervised learning strategy was developed to identify dominant click types in the training set based on click spectral shape and ICI distributions. The process consisted of two phases: The first phase automatically stepped through the recordings in five-minute increments (bins), and identified summary click characteristics (mean spectrum and modal ICI) for each bin. The second phase identified distinct, recurrent click types across all bins, producing template clusters for classification.

*Phase 1.* The purpose of the Phase 1 network was to identify consistent features of clicks within time bins. For each time bin, the set of all detected clicks in the bin was identified. To ensure that bins contained a representative sample, summary click types were produced for bins containing at least 100 click detections (click-positive bins). To reduce processing time, if a bin contained more than 5000 clicks, a randomized subset of 5000 clicks was selected for analysis. The size of the subset was chosen based on computation speed (pairwise click comparison has time complexity of order O(*n^2^*)). This reduction affected between 0.1 and 10% of click-positive bins, depending on the deployment (Table 2).

Click spectra (*u*) in dB re 1 μPa were truncated beyond 10 and 70 kHz, and received spectral levels of each click were normalized between [0, 1] as

$$u_{n}=\frac{u-\min\left(u\right)}{\max\left(u-\min\left(u\right)\right)}$$ (1)

where *u* is the vector of spectral levels of one click across the frequency range of interest, and *u*_*n*_ is the amplitude-normalized (indicated by subscript *n*) spectral level of that click.

The first difference (Δ*u*_*n*_) across normalized spectral bins was computed for each click spectrum. Pairwise similarity *D* was computed between the first difference of each pair of normalized spectral row vectors Δ*u*_*n*_ and Δ*v*_*n*_ using a correlation distance calculation [MATLAB pdist(); 41]:

$$D=1-\frac{(\Delta u_{n}-{\bar{\Delta u}}_{n}){\left({\Delta v}_{n}-{\bar{\Delta v}}_{n}\right)}^{\prime }}{\sqrt{(\Delta u_{n}-{\bar{\Delta u}}_{n}){\left({\Delta u}_{n}-{\bar{\Delta u}}_{n}\right)}^{\prime }}\sqrt{({\Delta v}_{n}-{\bar{\Delta v}}_{n}){\left({\Delta v}_{n}-{\bar{\Delta v}}_{n}\right)}^{\prime }}}$$ (2)

where ${\bar{\Delta u}}_{n}$ and ${\bar{\Delta v}}_{n}$ are the means of Δ*u* and Δ*v* respectively.

The distance between each pair of spectral vectors was converted into a similarity metric *S*_*SPEC*_ such that

$$S_{SPEC}=\exp\left(-D\right)$$ (3)

resulting in a matrix of edge weights in which all values are on the interval [0, 1] with weights closer to 1 indicating higher similarity between normalized spectra.

For each click-positive bin, a network was constructed in which nodes represented individual clicks, and edge weights were given by *S*_*SPEC*_. An undirected, non-pruned network of 5,000 nodes in which each node has been compared to all others contains 12.5 million (5,000^2^ / 2) edges. Many of these edges are weak and can be pruned from the dataset, reducing computation time without significantly affecting the information contained in the network [42, 43]. An exploratory analysis was conducted on a subset of the data (site MC deployment 1) to examine the effects of the amount of edge pruning (*p*_*e*_): *p*_*e*_ was varied between 0 and 0.99 (0 to 99% of weakest edges pruned). Effects of the pruning threshold are detailed in Results. Based on the exploratory analysis, *p*_*e*_ = 0.95 was chosen as a mid-range threshold. After pruning at this threshold, a 5,000 node network would contain 625,000 edges. Any weakly-connected nodes isolated from the network by pruning were excluded from further analyses.

In the pruned network, clusters of similar nodes were identified using the CW clustering algorithm [20], an approach often used in Natural Language Processing. CW is an agglomerative (bottom-up) clustering algorithm aimed at rapidly partitioning large networks. Each node in the network was initially assigned to its own category. Nodes were then iteratively re-assigned to the category of the nodes to which they were most strongly connected. Iterations continued until reassignments ceased, up to a maximum of 20 iterations. The CW algorithm has the advantage of being fast for large networks (speed scales linearly with number of nodes), and it was able to identify clusters of nodes that were very different in size, preventing small but significant clusters from being overshadowed by large clusters. Mean spectral levels were computed for all clusters consisting of 100 or more nodes. ICIs were computed as the first time difference between sequential clicks in each cluster and binned between 0.01 and 0.5 sec in 10 ms increments. Modal binned ICI values were computed for each cluster. In bins where dense clicking resulted in saturation at low ICIs, modal ICI was identified as the first peak in the ICI distribution. Mean spectral levels and modal ICIs were retained as “summary nodes” for input into Phase 2.

*Phase 2.* Summary nodes from Phase 1 were used to generate a second network in Phase 2. The purpose of this second network was to identify recurrent click types across many bins. Some sites had more click-positive bins than others, and therefore more summary nodes. To ensure that sites were evenly represented, a randomized subset of 1000 summary nodes were chosen from each of the five sites, for a total of 5,000 nodes.

A combined similarity metric (*S*_2_) consisting of both spectral and ICI information was computed, to allow both pieces of information to influence the relationships within the Phase 2 network. Spectral similarities (*S*_*SPEC*_) were computed as in Phase 1. ICI distances (*D*_*ICI*_) were computed as the Euclidean distance between modal ICI values. These distances were converted to a similarity (*S*_*ICI*_) metric using Eq 3. These two scores were then combined to produce *S*_2_ as

$$S_{2}=S_{ICI}∙S_{SPEC}$$ (4)

Like many agglomerative clustering routines, CW is non-deterministic because the starting node is selected at random. As a result, the composition of clusters can vary between trials. To identify a robust partition of the Phase 2 network, 20 independent runs of the CW clustering algorithm were performed (*p*_*e*_ = 0.95). Clusters containing at least 20 nodes were retained. After all iterations were complete, the normalized mutual information (NMI, [44]) criterion was used to assess the consistency of the Phase 2 partitions. NMI provides a measure between of cluster consistency across multiple trials on a [0, 1] scale, with higher NMI indicating more consistent cluster composition. NMI was computed between pairs of partitions *P*_*a*,_ *P*_*b*_ for *a* and *b* = 1,…,20 and *a*! = *b*. NMI was computed as follows for partition *P*_*a*_ consisting of *k*_*a*_ clusters containing $n_{i}^{a}$ nodes (*i* = 1,…, *k*_*a*_), and *P*_*b*_ consisting of *k*_*b*_ clusters with $n_{j}^{b}$ nodes (*j* = 1,…, *k*_*b*_):

$$NMI(P_{a},P_{b})=\frac{-2\;\sum_{i=1}^{k_{a}}\sum_{j=1}^{k_{b}}n_{ij}^{ab}\;\log\;\left(\frac{n_{ij}^{ab}∙n}{n_{i}^{a}{∙n}_{j}^{b}}\right)}{\sum_{i=1}^{k_{a}}log(\frac{n_{i}^{a}}{n})+\sum_{j=1}^{k_{b}}\log\;\left(\frac{n_{j}^{b}}{n}\right)}$$ (5)

The partition with the highest average NMI across all comparisons was chosen as the final partition (“Best of K” consensus clustering, [45]). The final partition *P*, consisting of *m* click type clusters *T*, was retained for use in classification.

### Cluster-based classification

The set of summary nodes identified using in the training set were used to automatically classify clicks in the test dataset (Table 2). As in the classifier training, Phase 1 of the automated clustering routine was executed on click-positive bins from test data to produce a set *C* of *n* test summary nodes spanning each test deployment. To classify each test summary node *C*_*i*_ in *C* (for *i* = 1 to *n*) from the test data to one of the click type clusters *T* from the training data, the spectrum and modal ICI of the test node was compared to all of the training nodes in each click type *T*_*j*_ of *P*, (for *j* = 1,…, *m*), to obtain a similarity metric following similar methods as for Phase II described above. The set of similarity scores was pruned, and *C*_*i*_ was automatically assigned to the cluster *T*_*j*_ with the highest mean similarity score between the test and training summary nodes.

The mean similarity between *C*_*i*_ and its matching cluster *T*_*j*_ was retained as a metric of classification certainty. In this classification exercise, the goal was to find the best click type match for *C*_*i*_, even if *C*_*i*_ was a poor quality example (e.g. noisy or sparse) so a lower *p*_*e*_ threshold (*p*_*e*_ = 0.90) was used to allow matching across a range of qualities by retaining more edges. Note also that this method of fusing spectral and ICI similarity scores using a product requires both scores to be strong in order to produce a strong match. Implications of this approach are further explored in the discussion.

To evaluate classifier performance, a systematic random sample of 200 test summary nodes from each site were manually assigned to a template cluster by a trained analyst reviewing mean spectra and ICI distributions of the test nodes. Test nodes that were not clearly similar to any of the click type clusters were labeled “unknown” by the analyst and counted as disagreements. The manual classifications were then compared with the automated classifications to evaluate classification confusion and to examine the relationship between automated classifier certainty and agreement between automated and manual classifications. Based on the evaluation, a minimum certainty threshold of 0.3 was established for automated classification. When evaluating classification confusion from the test subset, test summary nodes identified as unknown by either the manual or automated method were considered mismatches. Total detection rates of each click type at each site were evaluated for the full test set.

#### Towed hydrophone array recordings

Towed hydrophone array recordings were reviewed to identify high quality, low noise, visually-confirmed single-species encounters. A representative encounter was selected for pantropical spotted dolphin (*Stenella attenuata*), Atlantic spotted dolphin (*Stenella frontalis*), pilot whale (*Globicephala* sp.) and Risso’s dolphin (*Grampus griseus*) (Table 3). This was a preliminary analysis to identify possible matches between click types recorded in the presence of known species and click types recorded on the HARPs. The towed array dataset was processed independently from the seafloor instrument data. It is important to note that these recordings were collected along the US Atlantic coast and near the sea surface, therefore comparisons with the HARP recordings may be impacted by geographic variations and differences in sound reception near the sea surface. Future work will target collecting additional towed array data in the GOM for more robust comparisons.

Delphinid clicks were detected in towed hydrophone array recordings using the same detection method applied to HARP recordings. Clicks were localized using time difference of arrivals (TDOAs) between the two recording channels to obtain bearings of the click source relative to the vessel. An analyst reviewed localizations to identify high quality encounters with clear animal tracks. Localized clicks that were retained for clustering to reduce the inclusion of false positive detections associated with vessel and flow noise. Mean click spectral levels and ICI distributions were automatically computed for each single species encounter from the selected hydrophone array data using Phase 1 of the automated clustering method used for seafloor-sensor recordings with *p*_*e*_ = 0.95.
